# Dispersive Solid Phase Extraction of Melatonin with Graphene/Clay Mixtures and Fluorescence Analysis in Surfactant Aqueous Solutions

**DOI:** 10.3390/molecules29112699

**Published:** 2024-06-06

**Authors:** Lucía Gutiérrez-Fernández, Ana M. Díez-Pascual, María Paz San Andrés

**Affiliations:** 1 Universidad de Alcalá, Facultad de Ciencias, Departamento de Química Analítica, Química Física e Ingeniería Química, Ctra. Madrid-Barcelona Km. 33.6, 28805 Alcalá de Henares, Madrid, Spain; lucia.gutierrezf@uah.es (L.G.-F.); am.diez@uah.es (A.M.D.-P.); 2 Instituto de Investigación Química Andrés M. del Río (IQAR), Universidad de Alcalá, Ctra. Madrid-Barcelona Km. 33.6, 28805 Alcalá de Henares, Madrid, Spain

**Keywords:** melatonin, graphene, clays, dispersive solid phase extraction, fluorescence, surfactant

## Abstract

In this work, the dispersive solid phase extraction (dSPE) of melatonin using graphene (G) mixtures with sepiolite (SEP) and bentonite (BEN) clays as sorbents combined with fluorescence detection has been investigated. The retention was found to be quantitative for both G/SEP and G/BEN 4/96 and 10/90 *w*/*w* mixtures. G/clay 4/96 *w*/*w* mixtures were selected to study the desorption process since the retention was weaker, thus leading to easier desorption. MeOH and aqueous solutions of the nonionic surfactant Brij L23 were tested as desorbents. For both clays and an initial sample volume of 25 mL, a percentage of melatonin recovery close to 100% was obtained using 10 or 25 mL of MeOH as desorbent. Further, using a G/SEP mixture, 25 mL as the initial sample volume and 5 mL of MeOH or 60 mM Brij L23 solution as the desorbent, recoveries of 98.3% and 90% were attained, respectively. The whole method was applied to herbal tea samples containing melatonin, and the percentage of agreement with the labeled value was 86.5%. It was also applied to herbal samples without melatonin by spiking them with two concentrations of this compound, leading to recoveries of 100 and 102%.

## 1. Introduction

Melatonin (*N*-acetyl-5-methoxytryptamine), a derivative of the essential amino acid tryptophan, is a hormone secreted by the enigmatic pineal gland in response to darkness. It has numerous roles related to circadian rhythm control, and it directly influences metabolism, the immune system, and the somnus [1]. It has also been proved that melatonin alleviates neurodegenerative illnesses such as Alzheimer’s and Parkinson’s [2] and has anti-inflammatory effects and anticancer properties [3]. In addition, it is a powerful antioxidant agent; it can scavenge free radical species (both reactive oxygen species (ROS) and reactive nitrogen species (RNS)) and stimulate the activity of antioxidant enzymes [4]. Outside the Animal Kingdom, melatonin was first discovered in an alga in 1991 [5]. Since then, many studies have reported the presence of melatonin in plants in concentrations ranging from picograms to micrograms per gram of plant [6]. It has also been found in fruits, including grapes, apples, pineapples, tomatoes, bananas, and cherries [7], in olive oil, and in beverages such as wine. Given the importance of melatonin, accurate analytical methods together with suitable extraction protocols to determine it in fruits and plants are needed. Nonetheless, the analysis of melatonin in these samples is challenging due to the broad range of concentrations among the different fruit (or plant) types, the difficulty in choosing the appropriate extraction solvent, and the instability of melatonin owed to its strong antioxidant capability since it reacts speedily with other food components [4].

Melatonin can be detected by several methods, including radioimmunoassay (RIA) [8], enzyme-linked immunosorbent assay (ELISA), gas chromatography–mass spectroscopy (GS–MS) [9], and high-performance liquid chromatography (HPLC) with an electrochemical detector (HPLC–ECD) [10], fluorescence detector (HPLC–FD) [11], or coupled to mass spectrometry (HPLC–MS) [12]. These methods differ in their sensitivity and specificity. Chromatographic methods have been the most widely used for melatonin determination in recent years since they offer high sensitivity and excellent detection selectivity. Some results have been reported when analyzing the same fruit varieties using different detection methods [10,13]. The concentrations of melatonin found in Montmorency are 12.3 and 13–19 ng g^−1^, and in Balaton Tart Cherries, are 2.03 and 2.9 ng g^−1^.

Also, melatonin shows native fluorescence due to the presence of the indole group in its chemical structure, which makes it suitable for direct analysis by fluorescence. This technique has been widely used owing to its high sensitivity and selectivity, easiness, speediness, and low solvent consumption, and has been applied to different types of samples, such as pharmaceutical preparations and urine [14], biological [15], and food/beverage samples [16]. In the presence of cyclodextrins, the fluorescence of melatonin is enhanced [17], while reagents such as 2,3-naphthalenedialdehyde provide a fluorescent product of the reaction with melatonin, which is measured by absorbance and fluorescence [15]. Other systems, such as metal–organic frameworks (MOFs) encapsulated into molecularly imprinted polymers (MIPs) (MOF@MIP), have been utilized for developing a sensor with luminescence detection [16]. Thus, Zr-based MOF as nanoparticles were incorporated into the MIP to obtain a composite with the target analyte in its structure (the template). Upon extraction of the melatonin, a selective material for the molecular recognition of this compound was obtained.

Due to its low concentration (usually from picograms to micrograms) and serious matrix interferences in plants, foods, and biological samples, solid–liquid extraction (SLE) with Na_2_CO_3_ and diethyl ether has been typically used for the analysis of melatonin by HPLC in complex matrices. Further, SLE with HClO_4_ or acetone/methanol followed by solid phase extraction (SPE) in a C18 cartridge for purification and subsequent measurement via HPLC and ELISA immunoassay has also been applied [18]. SPE has been applied using different cartridges and solvents for elution [19]. On the other hand, melatonin in mulberry leaves has been determined via both SPE and liquid–liquid extraction (LLE) prior to its quantification by HPLC with fluorescence detection (HPLC-FD) [20]. Other solid phase extraction modalities have been developed, such as dispersive solid phase extraction (dSPE), which is based on the addition of a sorbent directly into the analytical solution followed by shaking, thus favoring the contact between the sorbent and the analytes [21]. Once the dispersion process is completed, the sorbent, with the analytes retained on its surface, is separated by a mechanical process, such as centrifugation or filtration. The most important advantage of dSPE is that the contact between the solid sorbent and the analyte is significantly more efficient than that of the SPE method. After the adsorption step, an appropriate solvent is needed for the analyte desorption. In this regard, the proper selection of the sorbent in SPE and dSPE is decisive to attain high selectivity. Many materials have been tested as sorbents, including MIPs, MOFs, mesoporous silica, and magnetic nanoparticles [22]. Carbon nanomaterials such as fullerenes, quantum dots (QDs), carbon nanotubes (CNTs), and graphene (G) have been used as sorbents in sample pretreatment [23] owed to their high specific surface area for analyte adsorption.

Graphene (G), a 2D carbon nanomaterial, has attracted significant attention due to its outstanding physical and chemical properties since it was first introduced in 2004 [24]. Its huge specific surface area and high chemical and thermal stability, combined with its electron-rich and hydrophobic properties, have made graphene an excellent sorbent for cleaning and preconcentration of target analytes [25]. Furthermore, G can interact strongly via π-π stacking interactions with compounds that contain aromatic rings owed of its large delocalized π-electron system. However, it is typically challenging to use G in this technique since it is an ultra-light material. In addition, it easily experiences irreversible aggregation in the packed cartridges during the SPE process, which would lead to reduced sorption capacity and extraction efficiency [26]. To address the abovementioned issues, novel approaches have been designed, such as the preparation of G-based magnetic materials [25] and the immobilization of G on the surface of silica [27].

Nanoclays such as sepiolite (SEP) and bentonite (BEN) are highly porous materials that have also been used as nanometric sorbents in dSPE. SEP (Si1_2_Mg_8_O_30_(OH)_4_(H_2_O)_4_·8H_2_O) is a hydrated magnesium silicate with a fibrous morphology, very high aspect ratio, and good mechanical properties, making it ideal for the development of hybrid materials [28]. Conversely, BEN (Al_2_H_2_Na_2_O_13_Si_4_) is composed mainly of the clay mineral smectite. Its structure comprises octahedral sheets of aluminum and tetrahedral sheets of silica, and it shows a layer-like or tissue-like texture. In previous studies, our research group has developed methods for the determination of riboflavin, polycyclic aromatic hydrocarbons, and tryptophan based on dSPE using sepiolite and mixtures of sepiolite and bentonite clays with graphene as solid sorbents [29,30,31]. In the present work, G/SEP and G/BEN mixtures at different weight ratios have been prepared in order to obtain nanomaterials with different polarities, and they have been used as sorbents for melatonin extraction prior to their determination by fluorimetry. Methanol, aqueous solutions of hexadecyl trimethyl ammonium bromide (CTAB) (a cationic surfactant), and polyoxiethylen-23-lauryl ether (Brij L23) (a nonionic surfactant) were used for melatonin desorption. The developed method has been successfully applied to the determination of this analyte in herbal tea samples in a fast and sustainable way.

## 2. Results and Discussion

The solid phase extraction sorbents used in this work (G/SEP and G/BEN mixtures) were prepared as described in the experimental section and were characterized by transmission (TEM) and scanning electron microscopy (SEM).

### 2.1. Electron Microscopy Analysis of Graphene/Clay Mixtures

**Figure 1:**
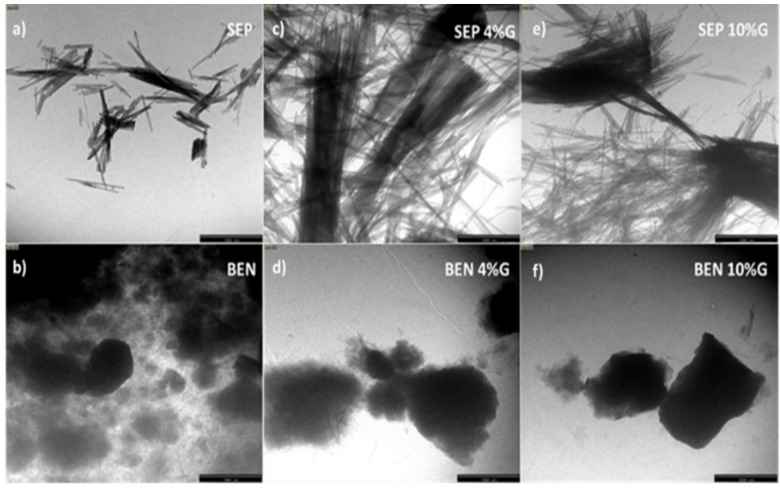
TEM images of neat SEP and BEN as well as G/clay mixtures with G percentages of 4 and 10 wt% at a magnification of 10,000×.

To obtain information about the surface morphology of sepiolite, bentonite, and their mixtures with graphene, the samples were observed by SEM and TEM, and representative micrographs are displayed in Figure 1 and Figure S1, respectively.

Regarding G/BEN mixtures, randomly alternated sheets of both compounds can be clearly observed. The most rigid platelets correspond to BEN, while the most flexible and thinnest sheets correspond to G. Also, good interaction between both compounds is suggested. In the mixture with 4 wt% G (Figure 1d), most of the flakes show diameters smaller than 30 nm since graphene intercalates within the bentonite lamellar structure, inducing the exfoliation of the flakes. The mixture with 10 wt% G (Figure 1f) shows less dense and more flexible structures, with many graphene sheets wrapping around the bentonite flakes, which are even thinner.

In the TEM image of pristine SEP, smooth, thin, rigid fibers can be observed (Figure 1a). They are aggregated in needles typically 50–100 nm in thickness. On the other hand, the TEM micrograph of neat BEN shows numerous rigid and quasi-spherical-shaped dark flakes (Figure 1b). Upon mixing with graphene, the sepiolite fiber bundles become thinner (Figure 1c,e), which is in agreement with the observations from the SEM analysis. Similarly, the bentonite flakes are more separated in the presence of graphene (Figure 1d,f), though in this case, it is more difficult to observe the graphene flakes since they are much lighter. Overall, microscopic observations reveal a uniform dispersion of both graphene and clay in the mixtures, thereby increasing their specific surface area, which is advantageous for application as sorbents in dSPE.

In the SEM images, pristine SEP fibers show a needle-like morphology, with sizes of 40–70 nm in diameter and >1 µm in length, Figure S1a. The fibers formed bundles-like aggregates by surface interaction between the hydroxyls (silanol groups, Si–OH) located at their external surface. Conversely, bentonite shows a lamellar structure composed of rigid individual nanoplatelets with thicknesses ranging from 30 to 50 nm (Figure S1b).

The images of the G/SEP mixtures show the graphene nanosheets randomly intercalated within the sepiolite nanofibers, suggesting a good interaction between both compounds. In the mixture with 4 wt% G (Figure S1c), the SEP nanofibers show diameters in the range of 30–50 nm, hence appear more separated than in pristine sepiolite due to the presence of graphene that inserts within the fiber aggregates and causes a debundling. This effect is even more pronounced in the sample with 10 wt% G (Figure S1e), in which more graphene flakes and fewer sepiolite fibers can be observed, and the fibers are much more separated.

### 2.2. Analysis of Melatonin in Different Media by Fluorescence

#### 2.2.1. Fluorescence Spectra of Melatonin in Different Media

**Figure 2:**
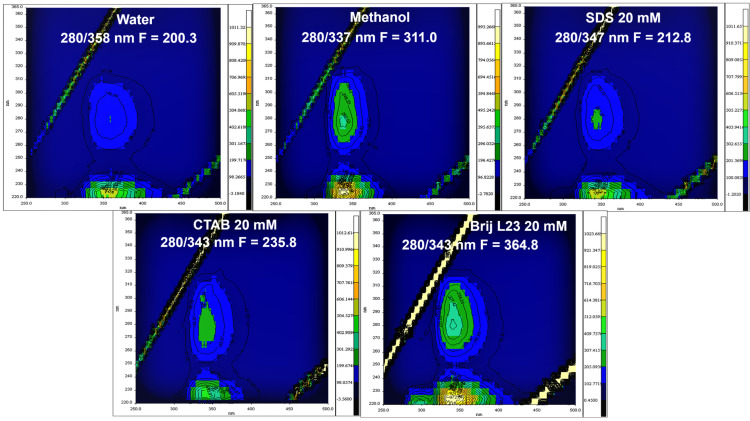
Fluorescence contour graphs of melatonin in water, methanol, and 20 mM aqueous solutions of SDS, CTAB, and Brij L23.

The fluorescence spectra of melatonin were recorded in different media, such as ultrapure water, for measuring the retention percentage and in methanol, sodium dodecyl sulfate (SDS), Brij L23, and CTAB as possible desorbents. Fluorescence contour graphs were obtained for a melatonin concentration of 80 µg L^−1^. The maximum excitation wavelength of melatonin is 280 nm for all the media studied, but the maximum emission wavelength depends on the medium. Figure 2 shows the fluorescence contour graphs of melatonin in the different media, namely water, methanol, and aqueous solutions of surfactants (SDS, CTAB, and Brij L23) at a concentration of 20 mM, which is above their critical micelle concentration (CMC).

As can be observed in Figure 2, the emission wavelength of melatonin in water is 358 nm, about 20 nm higher than that found in methanol (337 nm). This shift in the wavelength is attributed to the different polarity of the solvents. The higher polarity of water results in a higher emission wavelength due to the more polar microenvironment of melatonin in this solvent. The dipole moment of a fluorophore in the excited state is larger than in the ground state. Following excitation, the solvent dipoles can reorient or relax around the molecule dipole, which lowers the energy of the excited state. As the solvent polarity is increased, this effect becomes larger, resulting in emission at lower energies or longer wavelengths [32]. In the presence of surfactants, the fluorescence intensity changes depending on the interaction with micellar aggregates [33]. When the polarity of the molecule is low, it is highly soluble in the micelle aggregates, which results in a higher fluorescence intensity as occurs for pyridoxine and riboflavin and fat-soluble vitamins [34,35]. In the surfactant solutions, the emission wavelength of melatonin is between those of water and methanol, attributed to the interaction of melatonin with the aqueous micelles that leads to a more hydrophobic microenvironment than in water, but less than in methanol. Regarding the fluorescence intensity, the maximum value is observed in Brij L23 aqueous solution, followed by CTAB and methanol, while the minimum is found in water.

Given that the strongest fluorescence intensity of melatonin is achieved in the nonionic surfactant Brij L23, this medium was chosen to study the influence of the solvent concentration since the maximum fluorescence intensity (F) increases with increasing concentration. The fluorescence contour graphs of melatonin in 40, 60, and 80 mM Brij L23 are shown in Figure S2. As depicted in the figure, all the spectra are very similar, with a slight increase in F upon increasing surfactant concentration.

**Figure 3:**
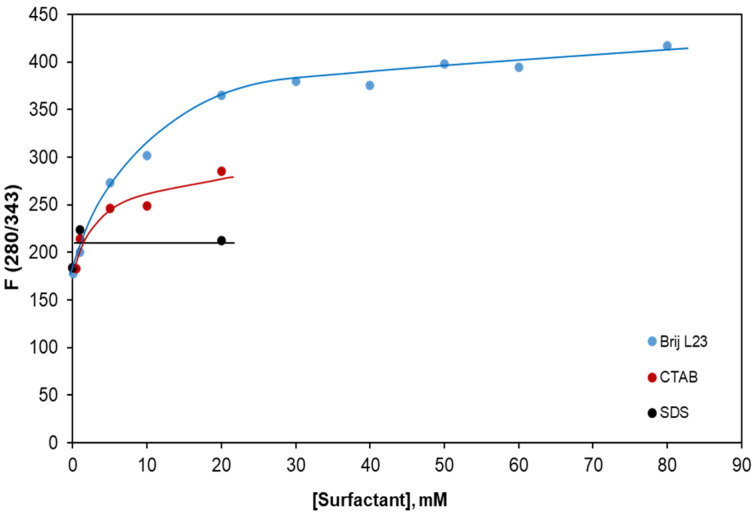
Change in the fluorescence intensity of melatonin as a function of the SDS, CTAB, and Brij L23 concentration. λ_exc_/λ_em_ = 280/343 nm.

The fluorescence intensity of melatonin 80 µg L^−1^ was recorded in 1 and 20 mM SDS, in 0.5, 1, 5, 10, and 20 mM CTAB, as well as in 0.05, 1, 5, 10, 20, 30, 40, 60, and 80 mM Brij L23. The excitation and emission wavelengths remain constant for all the concentrations of each surfactant. Figure 3 shows the change in melatonin fluorescence as a function of the surfactant concentration for excitation/emission wavelengths of 280/343 nm.

Regarding Brij L23, F rises by a factor of 1.98 from the value in the water solution ([Brij L23] = 0) up to a concentration of 20 mM and increases slightly at higher surfactant concentrations. In the case of CTAB, it continuously grows with increasing concentration, showing a 1.55-fold increase at a concentration of 20 mM. It is important to note that the CMC of Brij L23 is 0.09 mM while that of CTAB is about 10 times higher; therefore, the F value increases for both surfactants above the CMC. The fluorescence intensity of melatonin is significantly higher in Brij L23 solutions than in CTAB and SDS. The anionic surfactant leads to lower F values. Hence, it was discarded as desorbent in the following stages of this study.

#### 2.2.2. Analytical Characteristics of Melatonin Analysis in Water and Desorbents

The analytical characteristics of the fluorescence analysis of melatonin were investigated in water to determine the retention of melatonin in the G/C solids as the difference between the initial concentration and that in the supernatant after the retention process. For this purpose, four calibration curves were carried out using the external standard method. Table 1 shows the sensitivity, limit of detection, limit of quantification, robustness, and reproducibility.

Prior to determining the accurate recovery percentage of melatonin with the chosen desorbents (MeOH and Brij L23 solutions), the analytical characteristics of the fluorescence analysis in these media (MeOH and 60 and 80 mM Brij L23) were also determined and are shown in Table 1.

The sensitivity was found to be higher in 80 mM Brij L23, which is in agreement with the fluorescence contour graphs. For the three media, the limit of quantification is very low, ranging between 11 and 13 µg L^−1^. The robustness is systematically lower than 10% and lower than 5% for the two concentrations of Brij L23. Overall, the good analytical characteristics of the method corroborate its suitability for the analysis of melatonin.

A very good sensitivity and a low quantification limit have been obtained, allowing to determine melatonin concentrations higher than 12 µg L^−1^. Further, the robustness is acceptable (around 5% RSD).

### 2.3. Retention of Melatonin in Graphene/Clay Solid Mixtures

Melatonin retention was studied in two G/clay mixtures (4/96 and 10/90 *w*/*w*). Its initial concentration in aqueous medium was set as 80 µg L^−1^. The retention was found to be quantitative for both clays (SEP and BEN) combined with G (4/96 and 10/90 *w*/*w*).

The desorption of melatonin could be carried out using the two mixtures prepared herein since both show a retention percentage of 100%. However, the desorption is easier with the 4/96 *w*/*w* mixture since the retention is weaker due to its higher polarity. Therefore, this solid mixture was chosen to investigate the desorption process.

### 2.4. Desorption of Melatonin from Graphene/Clay 4/96 w/w Mixture with Different Desorbents

Melatonin desorption after retention in the 4/96 *w*/*w* mixture was carried out using three different media as desorbents: methanol (an organic solvent) as well as aqueous solutions of the cationic surfactant CTAB and the nonionic surfactant Brij L23. Both sepiolite and bentonite clays were used to prepare the solid mixture. The percentage of recovery was calculated by comparing the fluorescence intensity of melatonin in each desorbent with that measured initially (prior to the extraction process).

#### 2.4.1. Desorption with CTAB

Two CTAB solutions (10 and 20 mM) were used for the desorption of melatonin (80 µg L^−1^), and a recovery of 73% was attained for both concentrations in duplicate. Thus, the amount of melatonin desorbed from the solids hardly changes with increasing surfactant concentration. In addition, CTAB solutions are difficult to prepare due to solid precipitation below 20 °C.

#### 2.4.2. Desorption with Brij L23

**Figure 4:**
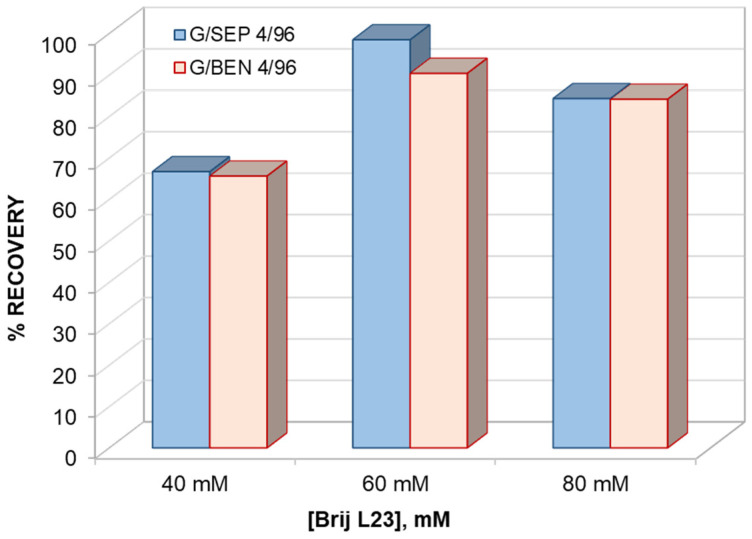
Percentage of melatonin recovery from G/clay (4/96 *w*/*w*) mixtures using different concentrations of Brij L23 as desorbent.

In order to study the potential of Brij L23 as a desorbent for melatonin, three different concentrations of this surfactant were tested (40, 60, and 80 mM), and the recoveries obtained are shown in Figure 4. The highest recovery (98.5%) was attained from the mixture comprising sepiolite clay using 60 mM Brij L23 as a desorbent. Thus, this surfactant solution has been chosen for the following experiments. Nonetheless, it should be noted that the recovery from mixtures comprising either SEP or BEN was also high (about 84.3%) for a surfactant concentration of 80 mM.

#### 2.4.3. Desorption with Methanol

The fluorescence intensity of melatonin in MeOH is slightly lower than that in Brij L23, though it is also high. The ability of this organic solvent to recover compounds in SPE is very useful due to its intermediate polarity; hence, the desorption with this solvent was carried out and compared with the surfactant solutions. The recovery was quantitative for both G/SEP and G/BEN (4/96 *w*/*w*) mixtures using an initial and final solvent volume of 25 mL.

#### 2.4.4. Comparison of the Extraction Recovery with MeOH, CTAB and Brij L23

The different media used for melatonin desorption were compared. The highest recovery attained for CTAB solutions (10 and 20 mM) was 73%, irrespective of the surfactant concentration. Further, as mentioned above, the recoveries were lower than those obtained with MeOH and Brij L23; hence, CTAB solutions were discarded as desorbents.

**Figure 5:**
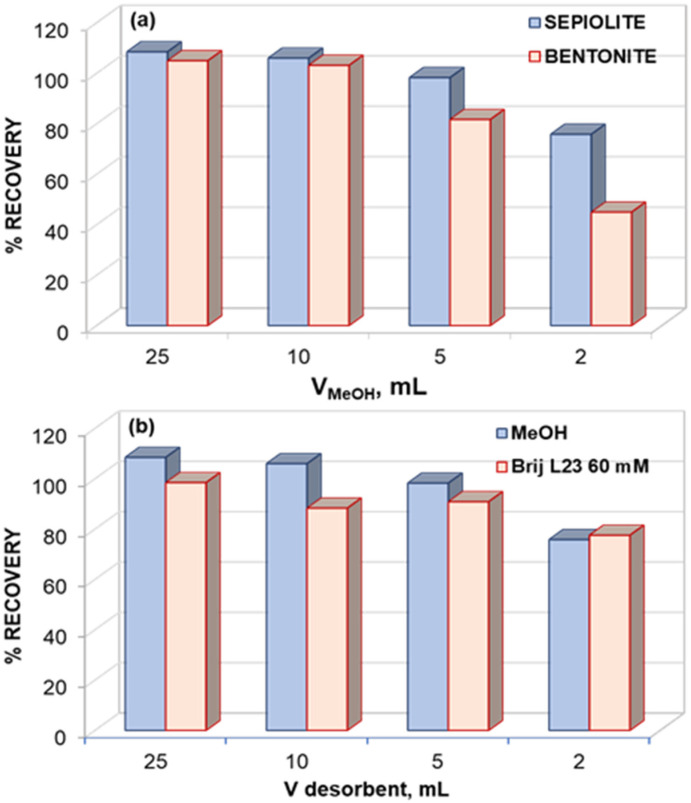
Percentage of melatonin recovery from G/clay 4/96 *w*/*w* mixtures using different volumes of MeOH as desorbent in sepiolite and bentonite (**a**) and using different volumes of MeOH and Brij L23 60 mM (**b**). Initial sample volume of 25 mL.

Both Brij L23 aqueous solutions and MeOH provide excellent recoveries, up to 98.3%, using 25 mL of desorbent. With the aim to preconcentrate the melatonin in the desorbent, lower volumes of MeOH were used. Figure 5a shows the recoveries from graphene mixtures with both clays, sepiolite, and bentonite, obtained with 25, 10, 5, and 2 mL of MeOH as desorbent. As can be observed, melatonin can be preconcentrated with the extraction process up to 5 and 2.5 times using sepiolite and bentonite mixtures, respectively.

In the desorption process, for MeOH volumes lower than 5 mL, the percentage of melatonin recovery decreases to 76% and 45% using 2 mL of SEP and BEN mixtures, respectively. A very high recovery (98.3%) from G/SEP (4/96 *w*/*w*) mixture has been attained using 5 mL of MeOH as desorbent, which implies a preconcentration factor of 5.

The percentage of recovery for different volumes (25, 10, 5, and 2 mL) of 60 mM Brij L23 solution as desorbent was comparatively analyzed (Figure 5b). For this purpose, G/SEP 4/96 *w*/*w* was also selected since this clay systematically provides better results than BEN. The recovery decreased slightly upon decreasing the desorbent volume to 78% for 2 mL. The comparison of the recoveries obtained for different volumes of MeOH or 60 mM Brij L23 as desorbents, using G/SEP 4/96 *w*/*w* mixture, is shown in Figure 5b.

The recoveries were high and quite similar for both desorbents. Since methanol is more dangerous than Brij L23, and the surfactant solutions are prepared in water, the most sustainable solvent, these were chosen to carry out the desorption process.

When the desorption volume decreased, the capacity of the desorbent remained unchanged up to a volume at which it could not desorb because of the impossibility of interacting with the molecules retained in the solid. Hence, it cannot solubilize the retained molecules. The process is a sum of different equilibria. On the one hand the interactions between melatonin and G/clay via π-π stacking between the aromatic rings and H-bond formation must be broken because of the stronger interaction between the desorbent and melatonin. In this regard, a higher desorption volume can solubilize the melatonin better (it gives a lower concentration), but in an analytical process, it is interesting to decrease the desorption volume in order to increase the preconcentration factor. Therefore, the influence of the desorption volume was studied, and the one that led to the lowest volume combined with the highest recovery was chosen. The best results were obtained for 25 mL of a sample volume in a G/SEP 4/96 *w*/*w* mixture as sorbent and 5 mL of 60 mM Brij L23 solution as desorbent. Hence, these were chosen as the optimal conditions for carrying out the experiments with real samples.

### 2.5. Analysis of Melatonin in Herbal Tea Samples

Melatonin content in herbal tea samples cannot be directly measured by fluorescence in aqueous solutions due to the interferences of the matrix. Therefore, the dSPE developed a method using a G/SEP (4/96 *w*/*w*) mixture as sorbent needs to be applied to efficiently separate the melatonin from the matrix prior to the fluorescence analysis. The results from the dSPE extraction combined with fluorescence measurement have been compared with those obtained with the dSPE method combined with a reference method (HPLC). This method was chosen as a reference since it can separate melatonin from other extracted compounds and determine its concentration, though it consumes high amounts of organic solvents such as methanol, which is not environmentally friendly.

Melatonin levels in the herbal samples were too high when an entire tea bag was used. Therefore, 8 mg of herbal tea bags were weighed and subsequently prepared as described in the experimental section. The limit of quantification calculated in the solid sample prepared in this way was found to be 0.4 mg g^−^^1^. The results obtained for both methods were compared. The standard deviations compared by the F test are statistically equal for a 95% confidence level (F = 0.63). For equal standard deviations, the comparison of the means using the Student’s *t*-test demonstrates the absence of statistical differences between them for the same confidence level and a *p*-value of 0.35. The amount of melatonin found in the herbal tea samples via fluorescence in the liquid extract is 50 ± 9 µg L^−^^1^, which corresponds to 1.6 ± 0.3 µg g^−^^1^ in the solid sample and is 44 ± 7 µg L^−^^1^ through HPLC, which corresponds to 1.4 ± 0.2 µg g^−^^1^. The amount labeled is 1.85 µg g^−^^1^. Therefore, the percentage of recovery using the fluorescence method considering the labeled value is 86.5%, which can be regarded as very high, taking into account the inhomogeneity of the sample.

Fluorescence measurements are faster, cheaper, and more environmentally friendly than HPLC. Hence, the developed method is a good alternative to determinate melatonin in real samples.

Another herbal tea sample without melatonin was chosen in order to demonstrate the accuracy of the developed method. This sample was spiked with two melatonin concentrations (8 and 10 µg L^−^^1^) below the LOQ of the fluorescence method (13 µg L^−^^1^). In this solid sample, from 5 mg of the sample extracted with 250 mL of water, the LOQ is 0.63 mg g^−^^1^.

The dSPE process combined with fluorescence allows the quantification of low levels of melatonin thanks to the desorption and preconcentration step using 10 mL of 60 mM Brij L23. To check for reproducibility, the experiments were carried out in quadruplicate on different days. For both spiked concentrations, the reproducibility is 5.0 and 5.9 as RSD for 8 and 10 µg L^−^^1^, respectively, which demonstrates the good precision of the developed method. The melatonin concentration found for the two concentrations added and the percentage of recovery using the whole method of analysis (dSPE extraction combined with fluorescence) is shown in Table 2. Results demonstrate the high accuracy of the method since the recovery is very high (100 and 102% for spiked concentrations of 8 and 10 µg L^−^^1^, respectively).

In these conditions (sample mass and water volume for SLE), the whole method can accurately determine melatonin as it has been explained above, but higher sample mass or lower water volumes may make it difficult to determine.

## 3. Materials and Methods

### 3.1. Reagents and Chemicals

Sepiolite (Mg_4_(Si_6_O_15_)(OH)_2_·6H_2_O, purity > 95%), a hydrated magnesium silicate with a very large surface area (close to 300 m^2^ g^−1^) comprising 60.2 wt% SiO_2_, 26.1% MgO, 1.7% Al_2_O_3_, and minor amounts of Fe_2_O_3_, CaO, Na_2_O, and K_2_O, was provided by Sepiol SA (Azuqueca de Henares, Spain). Few high-purity graphene layers in powder form, with a layer thickness smaller than 2 nm, a surface area close to 500 m^2^ g^−1^, and a low oxygen content (≤5 wt%), were supplied by Avanzare Innovación Tecnológica, SL (Logroño, Spain). Melatonin (C_13_H_16_N_2_O_2_, purity ≥ 98%, M_w_ = 232.3 g mol^−1^) and hexadecyltrimethylammonium bromide (CTAB), with a formula of C_19_H_42_BrN, critical micelle concentration of 0.9 mM and M_w_ of 364.5 g mol^−1^ were purchased from Merck (Darmstadt, Germany). Polyoxyethylene-23-lauryl ether (Brij L23) with a formula of C_12_H_25_(OCH_2_CH_2_)_23_OH, critical micelle concentration of 91 μM and M_w_ of 1198.6 g mol^−1^) and sodium dodecylsulfate (SDS), with a formula of C_12_H_25_O_4_SNa, M_w_ of 288.4 g mol^−1^, and critical micelle concentration of 8.3 mM) were provided by Sigma (Madrid, Spain). Methanol (CH_3_OH) with a density of 0.79 g cm^−3^ and M_w_ of 32.04 g mol^−1^ was provided by Scharlau (Barcelona, Spain). All the reagents were of high purity grade and used as received. Aqueous solutions were prepared in ultrapure water obtained from a Milli-Q system (Millipore, Milford, MA, USA). A stock solution of melatonin with a concentration of 20 mg L^−1^ was initially prepared, and the subsequent solutions were obtained by dilution and then stored in the dark at 4 °C. Herbal tea samples were obtained from a local store (Alcalá de Henares, Spain).

### 3.2. Instrumentation

Fluorescence spectra were obtained with an LS-50B fluorescence spectrophotometer (Perkin-Elmer, Hopkinton, MA, USA) incorporating a Xe lamp and thermostatised at 25 ± 1 °C with a Braun Thermomix BU bath (Analytical Instruments LLC, Minneapolis, MN, USA). For the measurements, quartz cuvettes with a conventional 1 cm path length were used at a speed of 500 nm min^−1^. The thickness of the excitation and emission slits was 5 nm, and FLWin Lab software was used for data treatment. A chromatographic system incorporating a Flexar binary LC Pump (PerkinElmer, Hopkinton, MA, USA) with vacuum degasification, a manual Rheodyne injection valve, 6-port, with a loop of 20 µL, and a Jet-Stream Plus thermostatic column (Knauer, Berlin, Germany), combined with a programmable fluorescence detector (Perkin-Elmer, Hopkinton, MA, USA) was used to perform the reverse-phase high-performance liquid chromatography (RP-HPLC) measurements. Chromatographic data acquisition and treatment were performed with TotalChrom WS software version 6.3.2 (Perkin-Elmer, Hopkinton, MA, USA). The analytical column, with dimensions of 5 µm, 150 × 4.6 mm, was a Chromaphase RP-18 provided by Scharlab (Barcelona, Spain). Measurements were performed using MeOH/H_2_O 50/50 *v*/*v* as the mobile phase at 25 °C and a flow rate of 1 mL min^−1^. The peak of melatonin was observed without any interference at a retention time of 3.5 min. pH measurements were performed using an InoLab pH meter (Mexico DF, Mexico). The mixtures were shaken with a mechanical stirrer (Selecta, Barcelona, Spain) and subsequently centrifuged with a refrigerated centrifuge (Digicen, Ortoalresa, Madrid, Spain). A UP400S ultrasonic probe (Hielscher Ultrasonics GmbH, Teltow, Germany), integrating a titanium sonotrode (ø = 3 mm; l = 100 mm) was used for sample ultrasonication. Transmission electron microscopy (TEM) micrographs at an amplification of 50,000× were obtained with a Zeiss EM-10 C microscope (Oberkochen, Germany) working at an acceleration voltage of 60 kV. Scanning electron microscopy (SEM) images at an amplification of 20,000× were acquired with a Zeiss DSM-950 microscope (Oberkochen, Germany), working at 15.0 kV. Statgraphics Centurion program, version XVII 17.0.16, was used for statistical analysis of the images.

### 3.3. Experimental Procedure

#### 3.3.1. Preparation of Graphene/Clay Mixtures

In order to attain materials with different polarities, several graphene/clay (G/C) mixtures (4/96 and 10/90 *w*/*w*, 100 mg) were prepared by weighing the necessary amounts of both solids and mixing them in 50 mL of distilled water. To attain homogenous dispersions, the mixtures were then subjected to sonication for 10 min with an ultrasonic probe at a power of 160 W, centrifuged for 5 min at a speed of 2598 g, and filtered with a cellulose filter (ø = 0.45 µm). The resulting solids were finally dried under ambient conditions and stored for the subsequent extractions. A diagram showing the entire procedure for the synthesis of the graphene/clay mixtures is shown in Figure S3.

#### 3.3.2. Scanning and Transmission Electron Microscopies

Both the raw clays and the different graphene/clay mixtures mentioned above were analyzed by transmission electron microscopy (TEM) and scanning electron microscopy (SEM). Regarding TEM analysis, a small amount of the solid sample was pulverized and suspended in water. Then, a drop of the dispersion was placed onto a cooper grid with carbon formvar and dried under ambient conditions afterward. With regard to SEM analysis, the pulverized samples were fixed onto a metallic sample holder with double-sided tape and then covered with a thin film of gold to prevent charging upon irradiation.

#### 3.3.3. Fluorescence Analysis of Melatonin Aqueous Solutions

Fluorescence contour graphs on melatonin (MEL) aqueous solutions with a concentration of 80 µg L^−1^ were registered to assess the fluorescence intensity (F) as well as the maximum excitation and emission wavelengths. MEL spectra were also registered in methanol and in aqueous solutions of CTAB and Brij L23 surfactants, which were explored as desorbing agents. The calibration curves in all the indicated media were recorded at the maximum excitation and emission wavelengths. The external standard method was applied to validate the developed methodology. The sensitivity, limit of detection (LOD), limit of quantification (LOQ), robustness, linear range, and precision of the method using Brij L23 and MeOH as desorbing agents were assessed using just the fluorescence method as well as together with the dSPE process. The sensitivity was obtained from the calibration curve as the ratio of the change in the concentration to the change in the fluorescence intensity. The LOQ and LOD were calculated as the concentration matching the intercept plus ten or three times the standard deviation of the intercept, respectively. The robustness was calculated as the relative standard deviation of the slopes of the calibration curve obtained on four different days. The intra-day precision (repeatability) and inter-day precision (reproducibility) were estimated, respectively, as the relative standard deviation of four measurements carried out within the same day or on different days.

#### 3.3.4. Extraction of Melatonin Using Graphene/Clay Mixtures as Sorbents

The extraction of MEL was first carried out by mixing 10 mg of the graphene/clay solid mixture with 25 mL of a melatonin solution at a concentration of 80 µg L^−1^. The mixture was agitated for 15 min and then centrifuged at a speed of 2598 g for the same period. Upon elimination of the supernatant, fluorescence measurements were performed at excitation and emission wavelengths of 280 and 340 nm, respectively. The retained MEL was calculated as the difference between the final and the first concentration measured. After solid isolation, the tube containing the solid was mixed with 25 mL of the solution containing the desorbing agent (MeOH, CTAB, or Brij L23), agitated for 15 min, and finally centrifuged under the identical conditions to those mentioned for the extraction stage. The desorption volume varied from 25 mL to 5 mL with MeOH and Brij L23, respectively. Finally, the supernatant was measured by fluorescence at the indicated excitation and emission wavelengths.

#### 3.3.5. Melatonin Extraction and Determination in Real Samples

Melatonin concentration in herbal tea samples was determined by fluorescence measurements after the dSPE process, and the results were compared with a reference method (HPLC). 8 mg of the herbal tea bags were treated with 100 mL of ultrapure water and heated for 10 min at the boiling point. The solution was filtered, and the dSPE method was applied using 25 mL as sample volume with 5 mL of desorption volume (preconcentration factor of 5). The supernatant obtained was separated and measured by fluorescence and HPLC. The results from both methods were compared by a statistical *t*-test comparison.

Another herbal tea sample without melatonin was spiked with low concentrations of the analyte. In total, 5 mg of the herbal tea were weighed, the appropriate volumes of a 20 mg L^−^^1^ melatonin stock solution were added to the solid, and they were treated with 250 mL of ultrapure water followed by heating for 10 min at the boiling point. The melatonin concentrations in the extract were 8 and 10 µg L^−^^1^. In total, 25 mL of the supernatant was extracted and desorbed with 5 mL of Brij L23 60 mM by dSPE and measured by fluorescence. The melatonin concentration was quantified after a preconcentration in the dSPE extraction step with 60 mM Brij L23.

## 4. Conclusions

In this work, an easy and cheap method for the direct determination of melatonin that combines a dSPE stage with fluorescence analysis has been developed. G/SEP and G/BEN 4/96 and 10/90 *w*/*w* mixtures have been tested as sorbents for the extraction step, and the retention has been found to be quantitative for the mixtures with both types of clays. Hence, both can be used in the dSPE process. The desorption was found to be easier for the 4/96 *w*/*w* mixtures. Hence, they were chosen to investigate the desorption process. The percentage of melatonin recovery in these mixtures was very high (>90%) using MeOH and 60 mM or 80 mM Brij L23 as desorbents, though it was slightly lower for G/BEN than for G/SEP. Very high recoveries and a preconcentration factor of up to 5 have been achieved using the G/SEP mixture as sorbent and MeOH or 60 mM Brij L23 as desorbents. The optimal extraction conditions were a G/SEP (4/96 *w*/*w*) mixture as sorbent and a 60 mM Brij L23 solution as desorbent. A preconcentration factor of up to 5 with very good recovery (90.9%) can be attained by reducing the final volume of the extract to 5 mL.

In herbal tea samples containing melatonin, the results obtained via dSPE extraction combined with fluorescence measurement and with the HPLC method were statistically equal. The percentage of agreement between the melatonin concentration obtained by the whole developed method and the labeled value is 86.5%. Another herbal tea sample without melatonin was spiked with two concentrations below the limit of quantification of the fluorescence method, leading to very high recoveries (100 and 102%) that demonstrate the accuracy of the method developed herein.

## Figures and Tables

**Table 1:** Analytical characteristics of the fluorescence determination of melatonin in methanol and Brij L23.

	Water	MeOH	Brij L23 60 mM	Brij L23 80 mM
Linear range, µg L^−1^	12–215	11–215	12–220	13–220
r	0.9996	0.9997	0.9996	0.9995
Sensitivity, L µg^−1^	2.35	3.61	3.78	3.89
Limit of detection (LOD), µg L^−1^	4 ± 2	3.2 ± 0.8	3.5 ± 0.4	3.9 ± 0.5
Limit of quantification (LOQ), µg L^−1^	12 ± 3	11 ± 3	12 ± 1	13 ± 2
Robustness, %RSD (*n* = 4)	5.27	8.02	1.45	2.76
Reproducibility, %RSD (*n* = 4) 80 µg L^−1^	4.15	3.24	3.10	1.58
Reproducibility, %RSD (*n* = 4) 120 µg L^−1^	3.05	3.88	3.45	1.81

**Table 2:** Melatonin concentration in herbal tea samples spiked with two concentrations below the LOQ of the fluorescence method, obtained using 60 mM Brij L23 as desorbent (*n* = 4).

[MEL] Added, µg L^−1^	[MEL] Found, µg L^−1^	% Recovery
8.0	8.0 ± 0.4	100
10.0	10.2 ± 0.6	102

## Data Availability

Data is contained within the article and Supplementary Materials.

## Supplementary Materials

The following supporting information can be downloaded at https://www.mdpi.com/article/10.3390/molecules29112699/s1, Figure S1: SEM images of different G/C mixtures with a magnification of 5000×. Figure S2: Fluorescence contour graphs of MEL in different concentrations of Brij L23. Figure S3: Preparation of Graphene/Clay mixtures for dispersive solid phase extraction.
