# Supplementary material for: Dispersive Solid Phase Extraction of Melatonin with Graphene/Clay Mixtures and Fluorescence Analysis in Surfactant Aqueous Solutions
Source: Molecules. 2024 Jun 6;29(11):2699. doi: 10.3390/molecules29112699 (PMC11173625; doi:10.3390/molecules29112699)
Supplement: Supplementary file 1 [file molecules-29-02699-s001.zip › molecules-3021797-supplementary.pdf]

DISPERSIVE SOLID PHASE EXTRACTION OF MELATONIN WITH GRAPHENE/CLAY MIXTURES AND FLUORESCENCE ANALYSIS IN SURFACTANT AQUEOUS SOLUTIONS

Lucía Gutiérrez Fernández[1], Ana María Díez-Pascual[1,2], María Paz San Andrés[1,2*]

[1]Universidad de Alcalá, Facultad de Ciencias, Departamento de Química Analítica, Química Física e Ingeniería Química, Ctra. Madrid-Barcelona Km. 33.6, 28805 Alcalá de Henares, Madrid, España (Spain).

[2]Universidad de Alcalá, Instituto de Investigación Química Andrés M. del Río (IQAR), Ctra. Madrid-Barcelona Km. 33.6, 28805 Alcalá de Henares, Madrid, España (Spain).

***Corresponding Author:** mpaz.sanandres@uah.es

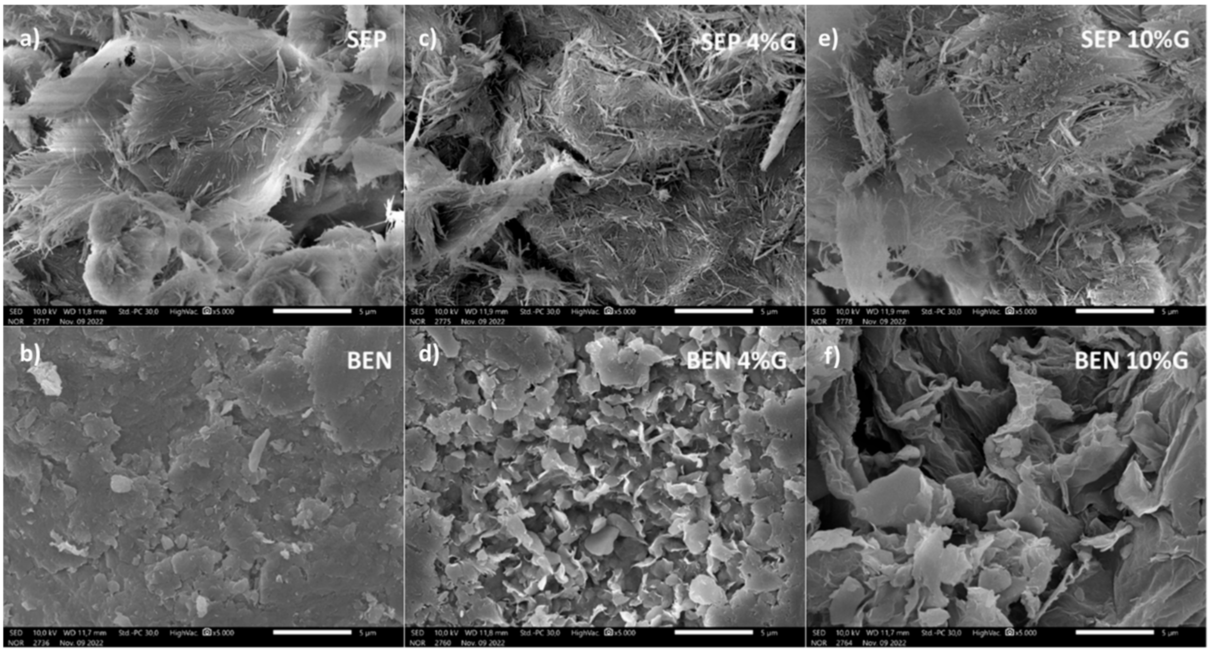

Figure S1. SEM images of neat SEP and BEN as well as G/clay mixtures with G percentages of 4 and 10 wt% at a magnification of 5000x.

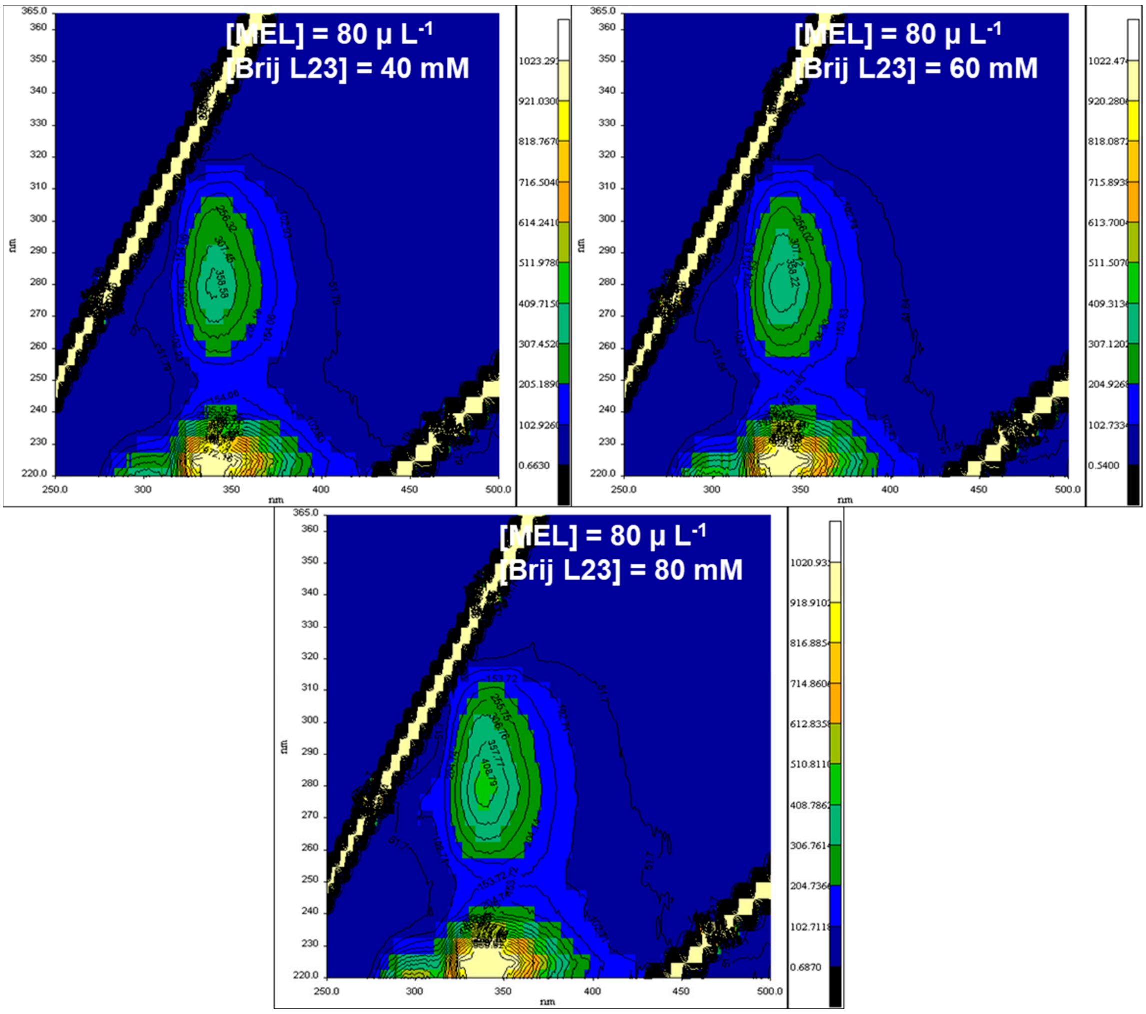

Figure S2. Fluorescence contour graphs of melatonin in different concentration of Brij L23.

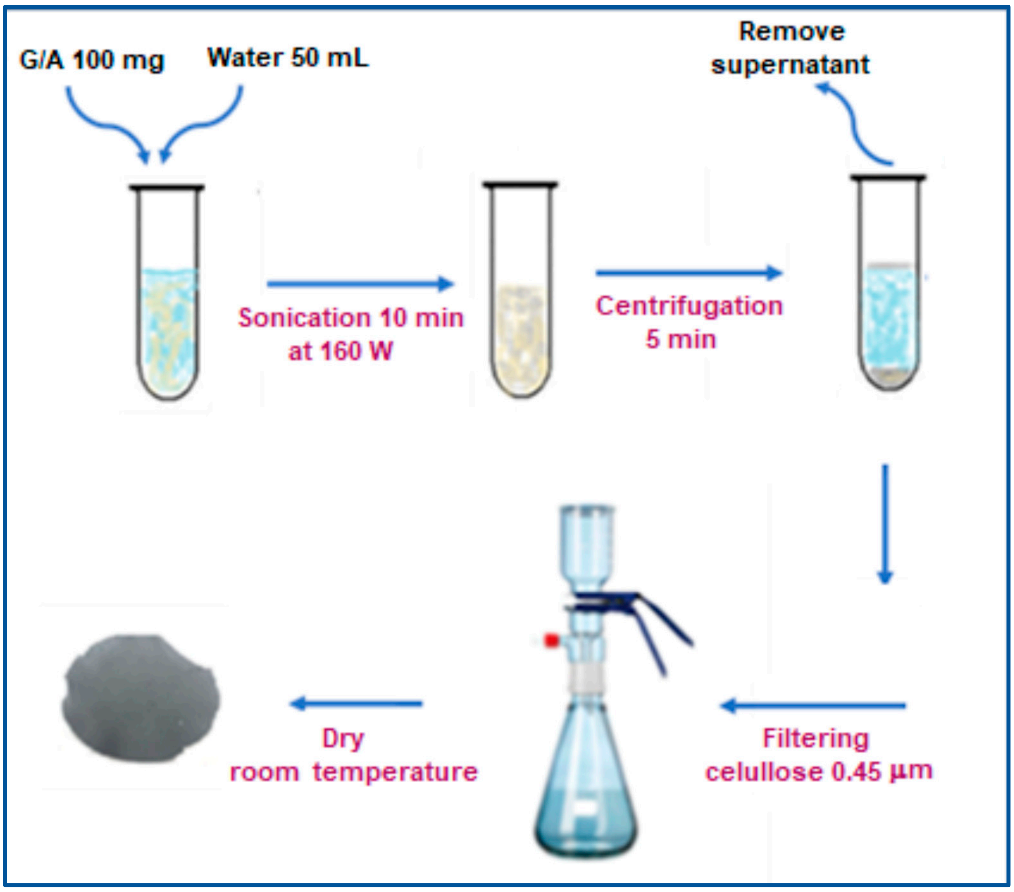

Figure S3. Preparation of Graphene/Clay mixtures for dispersive solid phase extraction.
